# Orchestrating HFpEF: How Noncoding RNAs Drive Pathophysiology and Phenotypic Outcomes

**DOI:** 10.3390/ijms262411937

**Published:** 2025-12-11

**Authors:** Angeliki Alifragki, Vasiliki Katsi, Konstantinos Fragkiadakis, Thomas Karagkounis, Nikolaos Kopidakis, Eleutherios Kallergis, Evangelos Zacharis, Emmanouil Kampanieris, Emmanouil Simantirakis, Konstantinos Tsioufis, Maria Marketou

**Affiliations:** 1 School of Medicine, University of Crete, 700 13 Iraklion, Greece; 2 1st Department of Cardiology, School of Medicine, National and Kapodistrian University of Athens, Hippokration General Hospital, 115 27 Athens, Greece; 3 Cardiothoracic and Vascular Surgery Working Group, Society of Junior Doctors, 151 23 Athens, Greece

**Keywords:** noncoding RNA, miRNA, HFpEF, fibrosis, cardiac hypertrophy, pathophysiology, long noncoding RNA

## Abstract

Heart Failure with Preserved Ejection Fraction (HFpEF) is an increasingly prevalent clinical syndrome that poses a significant public health challenge due to its complex pathophysiology, diagnostic limitations, and lack of effective therapies. Central to its development are multifactorial and interrelated mechanisms, including left ventricular diastolic dysfunction, myocardial fibrosis, inflammation, endothelial dysfunction, and cardiomyocyte hypertrophy. In recent years, noncoding RNAs (ncRNAs)—particularly microRNAs (miRNAs) and long noncoding RNAs (LncRNAs)—have emerged as critical regulators of these cellular and molecular pathways. This review outlines the major pathophysiological mechanisms underlying HFpEF and highlights the noncoding RNAs most extensively studied in this context. Several ncRNAs have shown promise as biomarkers for the diagnosis and prognosis of HFpEF, owing to their tissue specificity, stability in circulation, and involvement in disease-relevant pathways. However, their integration into routine clinical practice remains limited. Importantly, the regulatory functions of ncRNAs in HFpEF pathophysiology also position them as potential therapeutic targets. Early experimental studies demonstrate encouraging results, suggesting that ncRNA-targeted interventions may support the development of personalized treatment strategies tailored to the diverse clinical phenotypes observed in HFpEF.

## 1. Introduction

Heart failure with preserved ejection fraction (HFpEF) presents a clinically challenging and heterogeneous syndrome, accounting for roughly half of all heart failure cases worldwide [1,2]. Unlike heart failure with reduced ejection fraction, HFpEF is defined by a normal or near-normal left ventricular ejection fraction, yet patients experience similar symptoms such as exertional dyspnea, fatigue, and reduced exercise tolerance [3,4,5]. This syndrome is driven by a complex interplay of risk factors and comorbidities, including advanced age, female gender, hypertension, obesity, diabetes, chronic kidney disease, and atrial fibrillation, which intricately shape its diverse phenotypes and clinical manifestations [3,4,5]. The prevalence of HFpEF has increased over time, rising from 38% in the late 1980s to 54% in the early 2000s, and is expected to continue increasing due to population aging and the rising prevalence of comorbidities such as obesity. The annual incidence among adults is reported to be between 7 and 18 per 1000 persons per year, with HFpEF patients typically being older and more often female [1,2,6,7,8].

**Figure 1:**
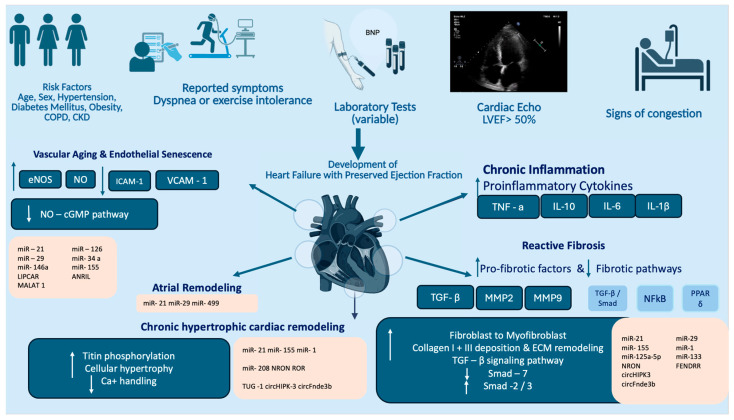
Overview of the main clinical and pathophysiological components involved in the development of HFpEF. Key clinical risk factors include advanced age, female sex, hypertension, obesity, diabetes mellitus, chronic kidney disease (CKD), and chronic obstructive pulmonary disease (COPD). Patients may present with fatigue, dyspnea, or progressive exercise intolerance; however, a subset may remain asymptomatic, with variable B-type natriuretic peptide (BNP) levels observed in laboratory testing. Echocardiography typically demonstrates preserved systolic function alongside possible signs of congestion. The figure also illustrates the principal pathophysiological mechanisms contributing to HFpEF and highlights associated noncoding RNAs implicated in each pathway. In this illustration, Arrows facing upwards and downwards are used to indicate increased and decreased expression of a molecule/pathway accordingly. (This illustration was created using the icon library of https://app.biorender.com/).

While HFpEF in Western countries is primarily associated with pre-existing risk factors such as diabetes mellitus and hyperlipidemia, in developing countries, arterial hypertension emerges as the predominant risk factor, with affected individuals typically being younger than those in Western populations [4]. The “skinny HFpEF” phenotype, characterized by lean, hypertensive patients with pronounced left ventricular hypertrophy, is more commonly observed in individuals of Asian descent. This contrasts with the predominantly obese HFpEF phenotype seen in Western populations [5]. Underlying mechanisms that account for HFpEF are usually related to aging, organ shape, and structural changes [8], as well as cardiometabolic shifts that persist in the cardiac tissue. At the mechanistic level, HFpEF is characterized by left ventricular diastolic dysfunction, increased ventricular and vascular stiffness, impaired myocardial relaxation, and often significant remodeling of the atria and pulmonary vasculature. Inflammation, oxidative stress, endothelial dysfunction, and disruptions in nitric oxide signaling are considered fundamental contributors to its pathogenesis and progression (Figure 1).

In recent years, noncoding RNAs (ncRNAs)—including microRNAs, long noncoding RNAs, and circular RNAs—have emerged as critical post-transcriptional regulators orchestrating molecular and cellular pathways implicated in HFpEF development and phenotype specification [9]. These noncoding RNA “maestros” influence cardiac hypertrophy, fibrosis, inflammation, and metabolic processes, holding promise as both biomarkers and therapeutic targets in HFpEF. This review synthesizes current understanding of HFpEF pathophysiology and phenotypic diversity, with a focus on the regulatory roles of noncoding RNAs and their potential for advancing mechanism-based diagnostics and treatments.

## 2. Current Pathophysiological Concepts on HFpEF

Heart Failure with Preserved Ejection Fraction (HFpEF) has unique pathophysiology and is quite different compared to Heart Failure with Reduced Ejection Fraction (HFrEF), which mainly occurs after acute myocardial injury following a myocardial infarction [7]. A variety of interconnected mechanisms, involving both cellular and extracellular components, contribute to the pathogenesis of the syndrome, for which effective management and treatment strategies remain only partially defined. The heart ages through progressive structural, functional, cellular, and molecular changes [8]. Key features include increased myocardial fibrosis, left ventricular wall thickening, impaired diastolic relaxation, and reduced compliance, all of which predispose to HFpEF [10]. Systematically orchestrated and long-standing inflammation enhances cardiac impairment with aging [11]. Cellular mechanisms increase oxidative stress, mitochondrial dysfunction, and impaired autophagy, resulting in damage and loss of cardiomyocytes, activation of fibroblasts, and altered calcium handling [10,12]. These changes result in stiffer myocardium and impaired ventricular filling, especially under stress or exercise. In the following paragraphs, we summarize the main pathophysiological events and involved pathways that occur systematically in the myocardium and vessels that predispose to the clinical phenotype of HFpEF (Figure 1).

### 2.1. Cardiac and Extracardiac Contributors in HFpEF

HFpEF arises from the interaction of intrinsic cardiac dysfunction, systemic comorbidities, and skeletal muscle abnormalities [13]. Cardiac contributors include diastolic dysfunction, ventricular stiffening, atrial remodeling, and pulmonary hypertension due to increased myocardial stiffness and impaired ventricular filling [14,15,16]. These changes are frequently exacerbated by atrial fibrillation and right ventricular involvement, which intensify symptoms such as exertional dyspnea and pulmonary congestion [17]. Systemic hypertension, obesity, diabetes, chronic kidney disease, and anemia act as key extracardiac factors, promoting low-grade inflammation and metabolic derangements that ultimately hinder peripheral perfusion and limit exercise capacity [18,19,20]. Furthermore, skeletal muscle dysfunction in HFpEF patients is characterized by muscle atrophy where fiber-type shifts towards glycolytic profiles, increased intermuscular fat, impaired perfusion, and mitochondrial dysfunction, all of which led to early fatigue and exercise intolerance [21,22,23]. Collectively, cardiac, extracardiac, and skeletal muscle features orchestrate the molecular and cellular pathways that, when disrupted, may result in HFpEF [24].

Notably, vascular aging plays a critical role in the pathophysiology of HFpEF and is characterized by structural changes in the vasculature that exacerbate cardiac dysfunction [11]. With advancing age, arterial stiffness increases due to extracellular matrix remodeling, collagen accumulation, and elastin degradation, resulting in elevated ventricular afterload and impaired left ventricular relaxation [25,26]. In addition, endothelial senescence, a hallmark of vascular aging, leads to reduced nitric oxide bioavailability, increased oxidative stress, and chronic low-grade inflammation, all of which promote coronary microvascular dysfunction and impair myocardial perfusion [27]. These vascular alterations contribute significantly to diastolic dysfunction and the overall progression of HFpEF.

Despite alterations seen on large vessels, capillaries do not remain untouched by aging. At the capillary level, vessels lose autoregulation with catastrophic results in metabolically demanding tissues like the brain, the heart, or the kidneys [11]. Those changes include endothelial distortion with pericytes’ loss, thickening of the basal membrane of the vessel, as well as limited NO (nitric oxide)-mediated vasodilation [11].

Emerging evidence also highlights the role of noncoding RNAs in regulating these vascular aging processes, linking molecular changes in the vasculature to cardiac impairment [28,29]. Understanding and targeting vascular aging mechanisms could open new therapeutic avenues to mitigate HFpEF progression and improve patient outcomes.

### 2.2. Molecular and Cellular Pathways Underlying HFpEF Development

The pathophysiological phenomena predisposing to HFpEF are regulated by various interconnected molecular pathways that are partially studied until the present day. At times acting synergistically and at other times counteracting one another, these pathways contribute to varying degrees to the manifestation of HFpEF phenotypes [30,31].

Natural aging is accompanied by endothelial dysfunction and can be further accelerated by conditions that compromise structural integrity. Endothelial function and vasodilation are mediated through the NO–cGMP (Nitric Oxide-cyclic Guanosine Monophosphate) signaling pathway. Normally, endothelial nitric oxide synthase (eNOS) catalyzes the production of NO from L-arginine. NO rapidly diffuses to adjacent smooth muscle cells and binds to the heme group of soluble guanylate cyclase (sGC), activating it to convert guanosine triphosphate (GTP) into cGMP [32]. When endothelial dysfunction is present, reduced NO bioavailability impairs sGC activity, and increased phosphodiesterase-mediated cGMP degradation further diminishes pathway efficacy [33]. As cGMP levels decline, the resulting signaling insufficiency leads to endothelial dysfunction that ultimately contributes to HFpEF progression. With aging, reductions in NO levels are anticipated, prompting cells to activate compensatory mechanisms. Initially, these include the upregulation of eNOS [34] and superoxide dismutase (SOD), a well-characterized enzyme with potent anti-inflammatory properties [35,36].

Besides insufficient vasodilation, impaired NO–cGMP-PKG (Protein Kinase G) axis impairs myocardial relaxation and enhances fibrosis of the myocardium, linking vascular aging with myocardial dysfunction [37]. Studies have shown that pharmacological inhibition of phosphodiesterase 2 (PDE2), which normally enhances NO/GC/cGMP signaling, reverses cardiac fibrosis in experimental heart failure models [38]. This effect is dependent on intact NO-sensitive guanylyl cyclase and is abolished by NO synthase inhibition, confirming that the anti-fibrotic benefit is mediated specifically through the NO/cGMP axis [38]. A different study of pharmacological NO enhancement presents improved exercise tolerance and diastolic function, despite the small sample under study [39]. Furthermore, NO deficiency allows for unopposed ROS (Reactive Oxygen Species) generation, as NO normally acts as an ROS scavenger and suppresses sources of oxidative stress such as NADPH (Nicotinamide Adenine Dinucleotide Phosphate) oxidases and mitochondrial dysfunction [39,40]. Those pathways and their components are the main target in HFpEF treatment nowadays, such as antioxidant treatments, Sodium-Glucose Cotransporter 2 (SGLT-2) inhibitors, IL-6 antagonists, and NO-enhancing agents, with favorable results reported in the literature [41,42].

Shifting focus, another pathway central to disease progression is interstitial and perivascular fibrosis, two types of reactive fibrosis commonly encountered in HFpEF [43]. Endothelial dysfunction provoked by increased ROS formation and diminished NO levels promotes vascular smooth muscle cell (VSMC) switching to activated fibroblasts mediating ECM (Extracellular matrix) remodeling [44]. In this proinflammatory state, endothelial cells express Vascular Cell Adhesion Molecule-1 (VCAM-1) and Intercellular Adhesion Molecule-1 (ICAM-1) [45], which activate the TGF-β/Smad (Transforming Growth Factor-beta) signaling pathway, a cornerstone in fibrotic development seen in HFpEF [46]. Concurrent inflammation and fibrosis increase TGF-β Smad signaling, which triggers crosslinking of ECM collagen and distorts the cytoskeleton by suppressing ECM degradation via inhibition of metalloproteases, mainly MMP-2 and MMP-9 [47]. Through these effects, the TGF-β/Smad signaling pathway is reinforced, resulting in unopposed myocardial stiffness and diastolic dysfunction. Activated RAAS (Renin–Angiotensin–Aldosterone) axis, frequently seen with aging, has been linked with the TGF-Β signaling pathway through elevated angiotensin II stimulating TGF-β expression [48]. Ongoing fibrosis triggers secretion of proinflammatory molecules like IL-6, IL-1β, and TNF-a (Tumor Necrosis Factor-a) locally and systematically with various consequences. For example, TNF-a has been linked with ECM remodeling and cardiomyocyte apoptosis when binding to the TNFR1 receptor, while the TNFR2 receptor exerts cardioprotective properties when binding the same molecule, as studies have shown [49]. Additionally, IL-6 mediates maladaptive cardiac hypertrophy through decreased phosphorylation of titin, and IL-1β increases metalloprotease expression, enhancing ECM turnover [50].

Metabolic disequilibrium and structural changes also play a contributory role in the initiation, maintenance, and progression of HFpEF. Hyperglycemia is another known regulator that drives fibroblast activation through the accumulation of advanced glycation end products cross-linking ECM proteins and further stimulation of TGF-β signaling [51]. Lipotoxicity, increased and modified epicardial fat, also regulates diastolic dysfunction in HFpEF [52]. In addition, long-standing atrial fibrillation is accompanied by advanced atrial remodeling under chronic atrial cell stretching, which is known to provoke proinflammatory stimulation and neurohormonal activation, both mechanisms implicated in HFpEF [53].

“Inflammaging” is a newly described term that connects the chronic exposure of the vasculature to activated inflammation. As previously mentioned, IL-1, IL-6, TNF-a, and other molecules exacerbate vascular aging, while silencing or pharmacologic blockage of those molecules in murine models restores endothelium-dependent vasodilation. This further emphasizes that anti-inflammatory strategies are quite promising tools in reversing vascular aging and possibly reverting HFpEF [54,55].

Although the molecular pathways underlying the pathophysiological mechanisms of HFpEF have been characterized sufficiently, the contribution of noncoding RNAs to these processes remains only partially explored. As key epigenetic regulators, noncoding RNAs hold significant potential in the context of HFpEF through direct and indirect regulation of cellular pathways and preexisting disease phenotypes.

## 3. Noncoding RNAs as Regulators in Cardiovascular Disease and HFpEF

In the diverse setting of cardiovascular disease, noncoding RNAs might play a discriminative role among syndromes like HFpEF, which have variable clinical phenotypes and quite variable outcomes in patients [56]. It is proven that noncoding RNAs actively participate in the regulation of cardiac hypertrophy, fibrosis, inflammation, and vascular function. All these mechanisms have different potential in causing HFpEF; therefore, unraveling those pathways may be helpful in disease modification.

In the majority of HFpEF cases, cardiac hypertrophy commonly develops as a result of chronic pressure overload and increased arterial stiffness with co-existing comorbidities such as hypertension and obesity, further enhancing concentric remodeling [15]. Local and circulating noncoding RNAs may influence the hypertrophy process in HFpEF through regulatory pathways like calcineurin/NFAT (Nuclear Factor of Activated T-cells), MAPK (Mitogen-Activated Protein Kinase), and PI3K-Akt (Phosphatidylinositol 3-kinase)/Protein Kinase B(Akt)) pathways [57]. *miR-133* directly represses calcineurin and NFAT signaling, as demonstrated by reciprocal regulation in cardiac hypertrophy models. Accordingly, *miR-133* downregulation leads to increased calcineurin activity and NFATc4 expression, while *miR-133* overexpression suppresses both, attenuating hypertrophic responses [58,59,60]. Additionally, *miR-133* modulates the PI3K-Akt pathway by targeting IGF1R and EGFR (Epidermal Growth Factor Receptor), leading to decreased Akt phosphorylation and activity, which impacts cell proliferation and hypertrophy [61]. Furthermore, microRNA-208 is a cardiac-specific microRNA encoded within the *Myh6 gene* (*Myosin Heavy Chain 6*) (*miR-208a*) and *Myh7* (*Myosin Heavy Chain 7*) *gene* (*miR-208b*), which plays a central role in the regulation of cellular pathways involved in hypertrophy in Heart Failure with Preserved Ejection Fraction (HFpEF). This sequence promotes hypertrophic growth by repressing negative regulators of muscle growth such as myostatin and thyroid hormone-associated protein 1, and it is sufficient to induce cardiac hypertrophy and pathological myosin heavy chain switching in response to stress [62]. Knockout or therapeutic inhibition of *miR-208a* prevents hypertrophy and pathological remodeling and improves cardiac function in models of pressure overload and hypertension-induced heart failure, which are mechanistically relevant to HFpEF [63]. Noncoding RNAs that are involved in myocardial hypertrophy are presented in Table 1.

Deposition of fibrotic tissue is a normal response to tissue damage, aimed at restoring function [64]. However, multiple injuries or recurrent vascular damage promote excessive fibrosis, distorting normal architecture and causing organ failure [64,65,66]. While replacement fibrosis occurs after myocardial infarction (MI) in HFrEF, HFpEF patients typically develop interstitial fibrosis, which activates myofibroblasts [67]. These myofibroblasts secrete large amounts of extracellular matrix and evade normal apoptosis, resulting in persistent matrix deposition, tissue stiffening, and impaired muscle relaxation, clinically observed as diastolic dysfunction [68,69,70]. On the cellular level, the main molecules involved in ECM remodeling are MMP-2 and MMP-9, which originate primarily from cardiac fibroblasts activated by the TGF-β/Smad signaling pathway [71,72]. *miR-21* is a well-described pro-fibrotic molecule that enhances fibroblast proliferation and promotes TGF-β [73]. This upregulation results further in increased Smad 2/3 phosphorylation and decreased Smad7 expression, which favor ECM remodeling. On the other hand, *miR-29* is considered antifibrotic, with studies related to decreased TGF-β/Smad signaling activation under *miR-29* regulation [74]. Alongside this, *miR-146* has also been characterized as an antifibrotic miRNA through suppression of the NFkB (Nuclear Factor kappa-light-chain-enhancer of activated B cells) signaling pathway, with clinical studies confirming expression of *miR-146* with mortality [75]. Further conformational studies suggest that in *miR-146* deficiency, the NFkB pathway is found activated with increased secretion of IL-1β and IL-18 [76,77,78]. Similarly, in the regulatory cascade of fibrosis, *lncRNAs* (e.g., *NRON*, *MALAT1*) act by either promoting or inhibiting fibrotic responses [79,80,81]. Furthermore, *miR-125a-5p* is reported to possess an antifibrotic role, with its overexpression improving cardiac function and limiting fibroblasts’ proliferation [82], as well as *circRNAs,* which also contribute to fibrosis development (Table 1).

**Table 1:** Noncoding RNAs in pathophysiological phenomena of HFpEF.

Type of Noncoding RNA	Name	Pathophysiological Mechanism	Expression in HFpEF—Disease State	Molecular Pathway	References
miRNAs	miR-21	Myocardial fibrosis Cardiac hypertrophy Atrial collagen deposition	upregulated	suppression programmed cell death AP-1 and TGF-β1 signaling MAP Kinase pathway	[83,84,85]
	miR-29	Myocardial fibrosis ECM remodeling	downregulated	PGC1α pathway	[86,87]
	miR-155	Cardiac hypertrophy and fibrosis	upregulated in hypertensive individuals	Increased expression associated with greater reductions in SBP following eplerenone treatment	[88,89]
	miR-125a-5p	Cardiac fibrosis	antifibrotic role	ETS-1/PDGF-BB signaling pathway	[90]
	miRNA-1	Cardiac hypertrophy Cardiac Fibrosis	downregulated	Inhibition of RasGAP Cdk9 fibronectin Rheb	[91,92,93]
	miRNA-133	Myoblast proliferation Cardiac fibrosis	downregulated	Akt signaling pathway β-adrenergic signaling pathways	[94,95,96,97]
	miR-208	Cardiac Hypertrophy	promotes cardiac hypertrophy in response to high pressure stress	TGF-β signaling	[98,99,100]
	miR-499	Cardiac Remodeling Cardiac contractility	variable	Akt and MAPK signaling pathways	[101,102,103]
Long ncRNAs	NRON	Cardiac hypertrophy Cardiac fibrosis	variable	Calcineurin/NFAT pathway	[104]
	ROR	Cardiac Hypertrophy	variable	Sponging to miR-133 Suppression of fibrotic gene	[105]
	FENDRR	Cardiac fibrosis	upregulated	Upregulated in fibrotic remodeling Depression SMAD3 signaling/miR-106b increased synthesis CTGF, and ACTA2	[106,107]
	CARMEN	Cardiac Differentiation	upregulated	Increased activation of fibrotic and hypertrophic gene networks	[6,108]
	TUG-1	Myocardial fibrosis Cardiac hypertrophy	upregulated	miRNA sponge to miR-29b-3p, miR-29c, miR-133b, miR-129-5p Depression tissue growth factor (CTGF), ATG7, and SMAD3 Activation CHI3L1	[109,110,111,112]
circular RNAs	circHIPK3 circFndc3b	Cardiac Hypertrophy and Cardiac fibrosis	-	Calcium handling and pro-fibrotic signaling.	[113,114,115]
	circIGF1R	Cardiac Fibrosis	-		

Abbreviations used in the table: RNA: Ribonucleic acid; HFpEF: Heart Failure with Preserved Ejection Fraction; miR: microRNA; AP-1: Activator Protein-1; TGF-β1: Transforming Growth Factor beta 1; MAP: Mitogen-Activated Protein; ECM: Extracellular Matrix; PGC1α: Peroxisome proliferator-activated receptor gamma coactivator 1 alpha; SBP: Substrate Binding Protein; ETS-1: ETS proto-oncogene 1; PDGF-BB: Platelet-Derived Growth Factor-BB; Cdk9: cyclin-dependent kinase 9; MAPK: Mitogen Activated Protein Kinase; NFAT: Nuclear Factor of Activated T-cells; CTGF: Connective Tissue Growth Factor; ACTA2: actin alpha-2; CHI3L1: Chitinase-3-like protein 1.

### 3.1. The Role of miRNAs in HFpEF Pathogenesis

Given the central role of noncoding RNAs in the pathophysiological processes associated with HFpEF, it is rational to focus on those molecules involved at various levels of the disease cascade—particularly tissue-specific ones such as *miR-30*, *miR-29*, *miR-26*, *miR-378*, *miR-143*, *miR-24*, and *miR-133*—which are among the most extensively studied due to their multifaceted roles and significant impact on cardiovascular disorders [116]. Noncardiac-specific molecules contribute to cardiac remodeling, a milestone in HFpEF development. Despite structural similarities of several miRNAs, their roles in HFpEF pathophysiology seem opposed.

*miR-21* regulates fibroblast proliferation and sustains fibrosis [73]. Its upregulation in post-MI fibrotic regions of the myocardium correlates with fibroblast activation through the TGF-β–mediated pathway, underscoring its link to fibrosis in HFpEF [73]. Through this pathway, downregulation of Smad-7 leads to increased expression of Smad-2 and Smad-3, both of which promote ECM production [73]. Although these molecules have been studied mainly in HFrEF, the underlying mechanisms strongly relate to HFpEF pathophysiology [117]. Additional studies have associated angiotensin II–driven cardiac hypertrophy with concomitant *miR-21* upregulation in pressure-mediated heart failure, a relationship that warrants further investigation in HFpEF given the aging population and high burden of comorbidities [118]. The role of *miR-21* in diabetic cardiomyopathy has also been reported, linking its upregulation to fibrosis through androgen receptor inactivation and suggesting another promising pathway relevant to HFpEF [119]. Furthermore, research by Pizzino et al. showed that postoperative mitral valve replacement is followed by variable degrees of left ventricular reverse remodeling and proposed a measurable biomarker to monitor this process. Elevated levels of peXOs (plasma exosomes) enriched with *miR-21* were associated with greater reverse remodeling compared to lower levels, highlighting the potential of this molecule as a prognostic marker [120].

In contrast to the pro-fibrotic effects mediated by *miR-21*, *miR-29* is a distinct noncoding miRNA with antifibrotic properties in the context of HFpEF [121]. Studies have shown that when *miR-29* is downregulated, the expression of fibrotic molecules such as fibrillin-1 and collagen increases, contributing to essential ECM remodeling [122]. Its antifibrotic actions are mediated through suppression of the TGF-β signaling pathway via TGF-β2 inactivation [123]. Recent research has further highlighted its significance in cardiac hypertrophy, demonstrating in vivo that *miR-29* expression correlates with clinically relevant measures such as intraventricular septal thickness in individuals with hypertrophic cardiomyopathy [124]. At the molecular level, *miR-29* upregulation suppresses the nuclear receptor *PPARD* (*Peroxisome Proliferator-Activated Receptor delta*) and atrial natriuretic factor, thereby functioning as a protective rather than a hypertrophic stimulus [125].

*miR-208a* is encoded within the *Myh6 gene* and is predominantly expressed in adult cardiac tissue. It is a key regulator of cardiac hypertrophy, fibrosis, and electrical conduction. Overexpression of *miR-208a* mediated through TGF-β pathway activation resulted in increased expression of collagen type I, enhancing cardiac fibrosis [126]. It is also related to the provocation of arrhythmias and the repression of negative regulators of muscle growth. *miR-208b*, in contrast, is encoded within the *Myh7* gene and is primarily upregulated during cardiac stress or pathological remodeling. Its expression is closely linked to the induction of β-myosin heavy chain during heart failure, but it does not independently drive hypertrophy or fibrosis to the same extent as *miR-208a* [127]. *miR-208b* is more involved in the regulation of myosin isoform switching rather than direct modulation of hypertrophic or fibrotic signaling pathways.

These contrasting roles highlight the need to clearly distinguish the functions of each molecule and to understand their potential interactions within molecular pathways. Such clarification is essential for developing precise therapeutic targets and minimizing off-target effects that may arise when modulating these molecules.

Firstly, *miR-143* acts via inhibiting adducin3 (add3), activating an F-actin capping protein, warranting proper cardiac contractility; therefore, its inhibition is related to contractility disorders [128]. Regeneration of the failing heart has been attributed to *miRNA-138* and *miRNA-195/miRNA-15* family, which are related to cardiac development during the early stages of life in several studies, and their role in HFpEF pathogenesis remains elusive [129,130]. Studies present that during a disease state, gene expression might shift from adult patterns to fetal gene expression as an attempt to adapt through remodeling [131,132].

In addition, *miR-1*, a muscle-specific microRNA [133], has been linked to increased expression of pro-hypertrophic and pro-fibrotic genes such as Ras GTPase-activating protein (RasGAP), Cyclin-dependent kinase 9 (Cdk9), and fibronectin (Table 1), leading to adverse cardiac remodeling and impaired relaxation of the left ventricle [91,92,93]. Interestingly, *miR-133* is produced via different reading of the same bicistronic transcript as *miRNA-1*, and it is known to enhance myoblast proliferation and inhibit differentiation by repressing serum response factor (SRF) and modulating β-adrenergic signaling pathways [134]. Reduced *miR-133* expression leads to derepression of pro-hypertrophic and pro-fibrotic signaling, resulting in maladaptive cardiac remodeling and increased stiffness, confirmed by many studies. Other extensively studied miRNAs are presented in Table 1 [83,84,85,86,87,88,89,90,94,95,96,97,98,99,100,101,102,103,135,136].

### 3.2. Other Noncoding RNAs in HFpEF

In the landscape of noncoding RNAs and their role in cardiovascular disease, microRNAs dominate the knowledge in both laboratory and research settings. Although evolving research has achieved significant milestones in the study of miRNAs, noncoding RNAs also have a significant role in Heart Failure with Preserved Ejection Fraction. Firstly, *fetal-lethal noncoding developmental regulatory RNA* (*FENDRR*) enhances fibrosis in the context of HFpEF [106,137]. *Cardiac Mesoderm Enhancer-associated Noncoding RNA* (*CARMEN*) orchestrates maladaptive remodeling manifesting ventricular dysfunction upregulated in HFpEF [108]. Clinical studies have tried to unlock the potential link between circulating levels of *FENDRR* and *CARMEN* with HFpEF. In the study by Marketou et al. [138], patients with hypertension and diagnosed Heart Failure with Preserved Ejection fraction were matched with hypertensive individuals with no overt signs of HFpEF, and levels of *FENDRR* and *CARMEN* were measured in plasma samples. Primarily, the study presented elevated levels of the two molecules in patients that have already developed a disease phenotype, i.e., HFpEF. Additionally, authors described a positive correlation between *CARMEN* and physical capacity estimated via Cardiopulmonary Exercise Test (CPET), while a weak negative correlation was attributed to *FENDRR*, underlying an important clinical tie-in of those molecules and exercise capacity [138]. *Taurine upregulated gene 1* (*TUG-1*) is a long noncoding RNA that, besides myocardial fibrosis, potentiates hypertrophy, apoptosis, autophagy, and inflammation with a confirmed role in the development of HFpEF through several studies [109,110,111,112].

Likewise, circular RNAs act as miRNA sequesters and participate in the development of HFpEF via direct or indirect contributory mechanisms in apoptotic or fibrotic pathways [139]. In HFpEF, *circRNAs* such as *circHIPK3* and *circFndc3b* have been shown to promote hypertrophy and fibrosis by interfering with calcium handling and pro-fibrotic signaling [113,114]. While others, such as *circIGF1R*, directly regulate cardiac fibroblast proliferation [115]. CircRNAs are highly stable and exhibit tissue- and disease-specific expression patterns, making them promising candidates for both diagnostic biomarkers and therapeutic targets in HFpEF.

### 3.3. Age- and Sex-Dependent ncRNA Regulation in HFpEF

Sex- and age-dependent differences exert a significant influence on how noncoding RNAs (ncRNAs) modulate the pathophysiology of HFpEF. Aging alters ncRNA expression profiles associated with cardiac aging and diastolic dysfunction, thereby contributing to the clinical and molecular heterogeneity of HFpEF. For example, the age-associated microRNA *miR-34a* is upregulated in aged mice and human myocardial biopsies [140]. Through downregulation of its target *PNUTS*—an anti-apoptotic regulatory gene—*miR-34a* enhances cardiomyocyte apoptosis via modulation of the checkpoint kinase-2 pathway and TRF2-related telomere-shortening mechanisms, processes known to reduce cellular lifespan [141,142]. Other studies have identified additional aging-related miRNAs, such as *miR-22*, which increases in cardiac fibroblasts from older mice [143], as well as *miR-18* and *miR-19*, which attenuate pro-fibrotic signaling by modulating TGF-β activity—the central driver of interstitial fibrosis in HFpEF [144].

Beyond aging, sex also plays a fundamental role in shaping ncRNA expression patterns. HFpEF is more prevalent in women [145], particularly in post-menopausal populations, and presents with sex-specific molecular signatures and regulatory pathways that influence disease progression and outcomes [2]. Altered estrogen signaling and X-linked ncRNAs are key elements underlying these differences. For instance, *miR-92a* regulates angiogenesis, and its inhibition has been associated with improved cardiac function and reduced mortality in experimental models [146]. *miR-223*, located on the X chromosome, modulates glucose–transporter–type 4 (GLUT-4) expression in the context of type 2 diabetes mellitus and inflammation [147], linking it to microvascular dysfunction and maladaptive cardiac remodeling characteristic of HFpEF. Estrogen-responsive miRNAs—including *miR-30* and *miR-146a* [148]—further contribute to sex-specific HFpEF phenotypes. Estrogen-mediated downregulation of antifibrotic miRNAs such as *miR-29*, coupled with upregulation of pro-fibrotic miRNAs like *miR-21* and *miR-125,* promotes fibrotic remodeling and may underlie the higher prevalence of HFpEF in women [149]. These observations highlight the need for deeper mechanistic understanding of sex- and age-related ncRNA differences to enable more precise patient stratification and targeted treatment approaches. Ensuring balanced inclusion of both sexes in preclinical and clinical studies is increasingly recognized as essential for generating translational insights into HFpEF pathobiology [150].

## 4. Diagnostic Biomarkers in HFpEF: Focus on miRNAs and Other Noncoding RNAs

Traditionally, the investigation of HFpEF is performed through laboratory testing, cardiac imaging with echocardiography, and evaluation of patients’ symptoms (Figure 1). N-terminal pro-B-type natriuretic peptide (NT-pro-BNP) is a peptide that is produced by ventricular cells as pre-pro-BNP in the setting of cardiac dysfunction or increased wall tension, and it is further processed to pro-BNP, which is subsequently cleaved to the active BNP form and inactive NT-pro-BNP form found in circulating plasma [151,152]. Therefore, it is strongly associated with heart failure with either reduced or preserved ejection fraction, and it is routinely measured for diagnosis [153]. This biomarker has high sensitivity to detect heart failure but low specificity in this disease and may be falsely interpreted in several patients’ population, such as obese individuals or individuals with chronic kidney disease or atrial fibrillation, conditions that usually co-exist with heart failure [1]. Therefore, to counteract those drawbacks, novel biomarkers are needed to aid heart failure diagnosis.

In the modern medicine era, NT-pro-BNP combined with novel biomarkers may offer personalized diagnosis and enhanced risk stratification together with effective treatment [153,154]. In patients with controversial presentation, mid-regional pro-atrial natriuretic peptide (MR-pro ANP) is a natriuretic peptide with higher sensitivity and specificity for HFpEF compared to NT-pro–BNP [155]. In the absence of known ischemia, high-sensitivity cardiac troponins (hs-cTnT, hs-cTnI) may play a prognostic role in HFpEF as well, despite their well-known role in acute coronary syndromes [156,157]. Soluble suppression of tumorigenicity-2 (sST2) and Galectin-3 indicate the underlying presence of myocardial fibrosis and inflammation, respectively, with the latter being prognostically related to mortality and heart failure hospitalization [158]. While their specificity is limited, other biomarkers such as C-reactive protein (CRP) and TNF-α, which reflect systemic inflammation, may provide additional prognostic information and remain under research [159].

Noncoding RNAs hold promising value in providing personalized risk stratification and prognosis. A systematic review and meta-analysis performed by Parva Reza and colleagues, which analyzed quantitatively 29 studies aiming to target different expressed miRNAs in the context of HFpEF versus HFrEF, concluded that seven miRNAs were repeatedly expressed and studied in HFpEF individuals [160]. Specifically, *miR-424-5p* was found upregulated in contrast with *miR-206*, *miR-328-5p*, *miR-30c-5p*, *miR-221-3p*, *miR-375-3p*, and *miR-19b-3p*, which were found downregulated in the setting of HFpEF, yielding the notion that miRNAs can also effectively distinguish between HFrEF and HFpEF [160]. In this context, studies evaluating prognostic properties of miRNAs compared to traditional biomarkers in patients with HFpEF and HFrEF are conducted. In the study by Zhang et al., *miRNA-19–b* was found to be lower in patients with HFrEF compared to HFpEF and healthy controls, manifesting as a useful tool for distinguishing between HFpEF and HFrEF combined with NT-pro–BNP [161]. Moreover, *miRNA-21* is another potent biomarker used for diagnosis and progression of HFpEF [162], independently correlated with hypertension, and serves as an effective target for treatment by reversing fibrosis and hypertrophy in the context of HFpEF [163,164].

Besides micro-RNAs, circulating long noncoding RNAs have also been tested for diagnosis of heart failure. *MHRT* (*myosin heavy-chain–associated RNA transcript*) is a heart-specific lncRNA transcribed from the *Myh7* locus, abundant in healthy hearts and acting as a cardioprotective regulator against fibrosis [165]. Clinical studies, including Kontaraki et al. [6], link lower *MHRT* expression with worse exercise capacity (indicated by VE/VCO_2_ slope) in patients with HFpEF, highlighting its contribution as a prognostic tool. *NRON* is another suggestive biomarker involved [165] in cardiac hypertrophy and remodeling, processes central to HFpEF pathogenesis [104] with direct implications for disease severity [166]. *LIPCAR* (*long intergenic noncoding RNA predicting cardiac remodeling*) is another circulating molecule that is elevated in patients with heart failure, including those with HFpEF, and is associated with adverse cardiac remodeling and diastolic dysfunction [167,168]. Higher plasma *LIPCAR* levels correlate with increased New York Heart Association (NYHA) class, impaired renal function, and markers of cardiac stress such as NT-proBNP and high-sensitivity troponin T, but not with systemic inflammatory markers. In patients with HFpEF, higher levels predict increased risk of cardiovascular hospitalization and mortality, particularly in those without chronic kidney disease [169]. Additionally, *LIPCAR* is also detectable in plasma-derived extracellular vesicles, supporting its utility as a stable, non-invasive biomarker for risk stratification and disease monitoring in HFpEF [170]. Selected noncoding RNAs from human studies are presented in Table 2.

Subsequently, it is overt that the use of noncoding RNAs in clinical practice offers diagnostic benefits and personalized stratification and management [171]. Apart from these, noncoding RNAs have a plethora of good qualities as biomarkers. Firstly, they are highly stable and resistant to pH variations, long-term storage at room temperature, and repeated freeze–thaw cycles, eradicating all preservation inaccuracies and providing reliable results [172]. Importantly, the levels of many noncoding RNAs can be accurately detected, not only due to their high tissue specificity but also because they are often encapsulated in macrovesicles or exosomes or bound to RNA-binding proteins, which enhances their stability and detectability [173].

Ongoing clinical trials and registries attempt to indicate the dynamics of the biomarkers’ use in actual clinical settings. Firstly, the UK Heart Failure with Preserved Ejection Fraction Registry (UK HFpEF) is a prospective cohort that recruits patients with Heart Failure with Preserved Ejection Fraction (EF > 40%) with the aim to identify noncoding RNAs and other multiomic molecules after complete phenotyping and diagnostic testing to detect the potential use of biomarkers and implement them in clinical settings [174]. Moreover, a Paramount prospective analysis highlighted the use of collagen homeostasis molecules as markers of progression and severity of HFpEF, whereas baseline pre-treatment galectin-3 might have modified the response to treatment with the angiotensin receptor neprilysin inhibitor [175]. In the pharmaceutical industry, the use of a synthetic molecule antagonizing *miR-132* expression (*antimiR-132*) managed to reverse cardiac remodeling in a pig model of HFpEF [176]. Interestingly, the first human phase 1b double-blinded, placebo-controlled study investigating the safety and associated outcomes of *microRNA-132* inhibition in humans demonstrated that *CDR132L*, a synthetic antisense oligonucleotide targeting *miR-132*, was safe and well tolerated in patients with chronic heart failure [177]. *CDR132L* produced a dose-dependent and sustained reduction in plasma *miR-132 levels*, with effective target engagement at doses ≥ 1 mg/kg. Furthermore, it was evident that patients who received *CDR132L* at these doses exhibited a median 23.3% reduction in NT-proBNP, compared to a 0.9% increase in the placebo group, as well as significant QRS narrowing and favorable trends in cardiac fibrosis biomarkers. These findings suggest potential cardiac functional improvements and justify further clinical studies to confirm the beneficial pharmacodynamic effects of *miR-132* inhibition in heart failure [177].

**Table 2:** Noncoding RNAs as novel biomarkers in HFpEF: evidence from human studies.

Type of Noncoding RNA		Clinical Use	References
miRNA	miR-424-5p miR-206 miR-328-5p miR-30c-5p miR-221-3p miR-375-3p miR-19b-3p	Distinguish between individuals with HFpEF and HFrEF	[160]
	miR-19-b	Distinguish between individuals with HFpEF and HFrEF	[161]
	miR-21	Prognosis of HFpEF among hypertensive individuals	[162,163,164]
	miR-423-5p	Multi-microRNA panels to distinguish HFpEF from healthy controls	[178,179]
Long noncoding RNAs	MHRT	Marker of functional capacity in HFpEF patients Prognostic potential	[6]
	NRON	Prognostic biomarker in heart failure	[165]
	LIPCAR	Associated with adverse cardiac remodeling and diastolic dysfunction in HFpEF Predict risk of cardiovascular hospitalization and mortality	[167,168]

Abbreviations in the table: HFpEF: Heart Failure with Preserved Ejection Fraction; HFrEF: Heart Failure with Reduced Ejection Fraction; MHRT: myosin heavy chain associated RNA transcript; NRON: non-protein coding RNA; repressor of NFAT; LIPCAR: Long Intergenic noncoding RNA predicting cardiac remodeling.

## 5. Noncoding RNA-Based Therapies for HFpEF: Potential and Limitations

### 5.1. Preclinical Data of Noncoding RNAs in Developing HFpEF Therapies

The multiple pathways discovered in the development of HFpEF shape new perspectives but also generate novel challenges in disease treatment. Current management of HFpEF includes symptomatic relief, management of decongestion (use of loop diuretics to achieve euvolemia) [180], and comorbidities eradication. The latest addition of Sodium–Glucose Cotransporter–2 (SGLT-2) inhibitors in clinical guidelines shows favorable results on mortality and progress in patients with HFpEF, yet more effective and personalized strategies are essential to be discovered [1]. Moreover, the partial or non-response of HFpEF patients to therapies mainly developed for HFrEF [181,182] and the large phenotypic diversity among patients with HFpEF are the driving forces of developing research around noncoding RNAs [183].

In the research trajectory of developing treatments, several epigenetic-targeting agents (“epidrugs”)—including compounds that modulate noncoding RNAs without altering the primary DNA sequence—have been evaluated in animal models and, to a limited extent, in humans, with ongoing development in the HFpEF therapeutic landscape [184]. In addition to these emerging molecules, commonly prescribed heart failure medications have been investigated for their effects on ncRNAs’ expression. Casieri et al. (2020) reported that ticagrelor, a widely used purinergic receptor P2Y12 inhibitor, was associated with increased secretion of cardiac progenitor cell–derived exosomes enriched in anti-apoptotic heat–shock–protein 70 (HSP70), thereby conferring protection under hypoxic stress [185]. Although this study was conducted in vitro, the use of human myocardial-derived progenitor cells highlights a promising cellular medium for identifying additional therapeutically relevant molecules and pathways. Furthermore, co-administration of current HFpEF therapies may exert previously unrecognized epigenetic actions that warrant systematic investigation. Supporting this concept, Grune et al. demonstrated that the nonsteroidal mineralocorticoid receptor antagonist finerenone suppresses the pro-fibrotic *miR-21* in the context of noncoding RNA modulation and HFpEF [186].

Beyond pharmacological interventions, several natural components of the Mediterranean diet also exhibit regulatory effects on ncRNAs [184]. Sulforaphane, a bioactive compound found in cruciferous vegetables such as broccoli, has been shown to prevent vascular remodeling via modulation of the nuclear factor erythroid 2-related factor 2 (NRF2) pathway and attenuation of cardiac fibrosis [187]. Moreover, expression of endothelial adhesion molecules ICAM-1 and VCAM-1—typically elevated in vascular injury—may be reduced through sulforaphane-mediated suppression of the TNF-α signaling cascade [188]. Furthermore, *micro-RNA 210*, a molecule that under hypoxic conditions drives cellular proliferation, differentiation, and angiogenesis with a protective role against maladaptive cardiac remodeling, can be directly regulated by another natural compound [189]. Crocin, a natural plant-based substance with known antioxidative properties, managed to elevate levels of *micro-RNA 210* in rodents with heart failure, manifesting its cardioprotective properties [190]. Furthermore, epicardial adipose tissue (EAT), a powerful regulator of cardiometabolic functions in patients with HFpEF, secretes circular RNAs in various patterns that regulate cellular functions at multiple levels. Even though *hsa_circ_0005583* contributes to DNA repair and regulation of the cell cycle, its role in HFpEF treatment requires further investigation [191]. Another study [192] highlighted the use of *miRNA-199* and its regenerative effects in cardiac tissues of adult pigs having experienced myocardial infarction. Uncontrolled remodeling and viral-mediated delivery of this micro-RNA may have resulted in high death rates (70% of treated) in this study, highlighting the need for additional influences on those treatments besides the desired result of ameliorating heart failure [193].

Significant discrepancies exist between preclinical rodent models and human HFpEF, particularly in terms of pathophysiological mechanisms and therapeutic responses. Rodent models frequently fail to recapitulate the full spectrum of cellular and molecular features observed in human HFpEF, which can lead to limitations in the interpretation and clinical translation of findings. As recently highlighted, understanding interspecies differences—especially in mitochondrial biology and ncRNA-related pathways—is critical for optimizing the design and interpretation of preclinical studies and for developing targeted therapies with better efficacy in humans [194]. Continued efforts are needed to refine animal models and validate molecular targets in well-characterized human cohorts before ncRNA-based interventions can be broadly applied in the clinic.

In an actual clinical setting, no such treatment for HFpEF has been implemented until the present. Although patisiran, an RNA interference therapeutic agent delivered via lipid nanoparticles that targets transthyretin (TTR) messenger RNA in the liver, thereby reducing circulating TTR protein levels, is relevant to HFpEF in the context of transthyretin cardiac amyloidosis, where it can slow disease progression and improve functional status, it is not a general treatment for HFpEF [195].

### 5.2. Advantages and Limitations in Integration of Noncoding RNA Therapies in Clinical Setting

The benefits given from the use of noncoding RNAs when used as treatment tools are prominent. Firstly, the abundance of noncoding RNAs in the human genome and their participation in various cellular pathways offer a great opportunity for HFpEF treatment, a highly diverse clinical condition [193]. Moreover, the circulating capacity of noncoding RNAs secreted via exosomes and other particles through the bloodstream underlines the feasibility of the therapies including those molecules [196].

Safety issues in integrating noncoding RNA (ncRNA) therapies into clinical settings include significant challenges with delivery, specificity, tolerability, potential toxicity, and unintended immunogenicity. Off-target effects are a major concern due to sequence similarities with endogenous RNAs, which can lead to unwanted gene silencing and adverse outcomes [197]. Additionally, the risk of overdosing or excessive expression may result in unforeseen side effects, including cancer or immune activation [198]. The pleiotropic nature of ncRNAs—where a single molecule can influence multiple cellular pathways—further complicates safety assessment and demands comprehensive investigation before wide clinical application. Robust patient monitoring, careful selection of delivery methods, and continuous refinement of therapeutic design remain essential to mitigate these risks and improve safety profiles.

## 6. Conclusions

The heterogeneity and complexity of HFpEF are key factors contributing to its rising prevalence. Its multifactorial pathophysiology—including aging, chronic inflammation, unchecked fibrosis, and remodeling—may precede clinical manifestation by years, often presenting initially as undiagnosed atrial fibrillation, diabetes, or obesity. Several modifiable (e.g., obesity, hypertension, and diabetes) and non-modifiable (e.g., age and sex) risk factors coexist with dysregulated expression of noncoding genomic regions, promoting disease progression.

What was once considered non-functional, untranslated genetic material is now recognized as a crucial regulator of molecular pathways involved in HFpEF pathogenesis. Among noncoding RNAs, microRNAs (miRNAs) and long noncoding RNAs (LncRNAs) have emerged as key players, although current knowledge remains fragmented. While some findings on their roles in fibrosis and hypertrophy are inconsistent, multiple studies consistently identify specific miRNAs in HFpEF, highlighting their potential as diagnostic biomarkers.

Treatment of HFpEF remains largely symptomatic. However, novel RNA-targeted therapies are under development, aiming to enable personalized management tailored to individual phenotypes. As developmental and pharmacological challenges are addressed, noncoding RNAs are poised to play a central role in ushering HFpEF treatment into the era of precision medicine.

## 7. Methods

This review aims to present the major pathophysiological pathways underlying HFpEF and highlight the most extensively studied noncoding RNAs, both as molecular regulators and as potential clinical biomarkers or therapeutic targets. Relevant literature was identified through a comprehensive search of PubMed, Google Scholar, and Scopus, focusing on studies published since 2000. The following keywords were used in various combinations: “noncoding RNAs,” “miRNA,” “HFpEF,” “Heart Failure with Preserved Ejection Fraction,” “fibrosis,” and “cardiac remodeling.” Articles not directly addressing the role of these pathways in HFpEF were excluded. Selected studies were analyzed thematically to synthesize current knowledge and identify emerging patterns or gaps in the literature.

## Data Availability

No new data were created or analyzed in this study.

## Abbreviations

The following abbreviations are used in this manuscript:

HFpEF	Heart Failure with Preserved Ejection Fraction
NO	Nitric Oxide
SOD	Superoxide Dismutase
eNOS	Endothelial nitric oxide synthase
cGMP	Cyclic guanosine monophosphate
PKG	Protein kinase G
SGLT-2	Sodium Glucose Transporter-2
SIRT	Sirtuin
VSMC	Vascular smooth muscle cell
VCAM	Vascular cell adhesion molecule
ICAM	Intracellular Adhesion molecule
TNF	Tissue Necrosis Factor
NFkB	Nuclear Factor kappa-light-chain-enhancer of activated B cells
TGF-β	Transforming growth factor-beta
FENDRR	FOXF1 Adjacent Noncoding Developmental Regulatory RNA
LIPCAR	Long intergenic noncoding RNA predicting cardiac remodeling
NYHA	New York Heart Association
